# 

**Published:** 1876-04

**Authors:** 


CONTENTS.

| | Page |
|---|---|
| The Physician | 145 |
| Surgical Diseases | 151 |
| The Aim of Life | 154 |
| The Modern Man | 156 |
| The Three Glories | 157 |
| Hearty Suppers | 157 |
| Cause of Consumption | 159 |
| Diarrhœa | 161 |
| Clerical Marriages | 162 |
| Material for Men | 165 |
| School Dangers | 166 |
| Eye Sight | 167 |
| Hydrophobia | 168 |
| Some Health Principles | 169 |
| Sleep of Children | 171 |
| Trouble Kills | 172 |
| A Wife Worth Having | 174 |
| The Marriage Relation | 175 |
| Winter Railroading | 176 |
| Gymnasiums | 177 |
| Neglecting Colds | 180 |
| Donation Parties | 182 |
| Public Schools | 182 |
| Cold Water Bathing | 186 |
| That Wicked Theft | 189 |
| Book Notice | 191 |

